# Kidney Tumor Semantic Segmentation Using Deep Learning: A Survey of State-of-the-Art

**DOI:** 10.3390/jimaging8030055

**Published:** 2022-02-25

**Authors:** Abubaker Abdelrahman, Serestina Viriri

**Affiliations:** School of Mathematics, Statistics and Computer Science, University of KwaZulu-Natal, Durban 4000, South Africa; 220106722@stu.ukzn.ac.za

**Keywords:** kidney tumor segmentation, deep learning, computerized tomography imaging, survey

## Abstract

Cure rates for kidney cancer vary according to stage and grade; hence, accurate diagnostic procedures for early detection and diagnosis are crucial. Some difficulties with manual segmentation have necessitated the use of deep learning models to assist clinicians in effectively recognizing and segmenting tumors. Deep learning (DL), particularly convolutional neural networks, has produced outstanding success in classifying and segmenting images. Simultaneously, researchers in the field of medical image segmentation employ DL approaches to solve problems such as tumor segmentation, cell segmentation, and organ segmentation. Segmentation of tumors semantically is critical in radiation and therapeutic practice. This article discusses current advances in kidney tumor segmentation systems based on DL. We discuss the various types of medical images and segmentation techniques and the assessment criteria for segmentation outcomes in kidney tumor segmentation, highlighting their building blocks and various strategies.

## 1. Introduction

**Figure 1:**
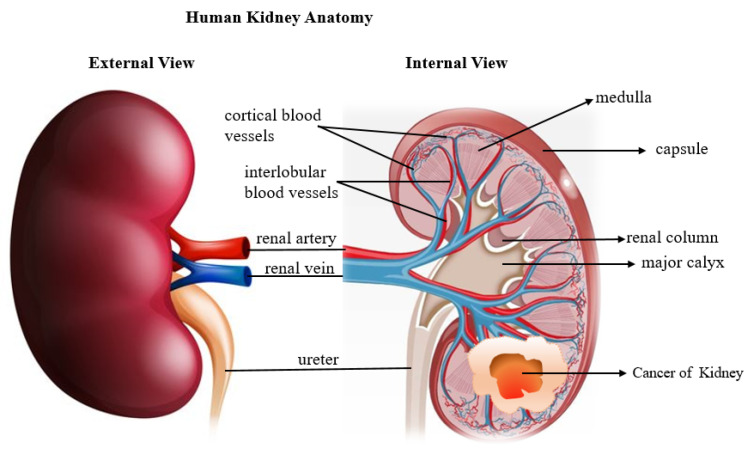
Diagram showing human kidney anatomy and Renal cell carcinoma developed inside the kidney.

The kidney is an organ of a vital role that keeps the body fluid and solute balance checked by excreting and filtering waste products. It also secretes many hormones and helps control blood pressure. The human kidneys are depicted in (Figure 1) [1]. Kidney cancer is one of the top 10 malignancies in men and women alike. The probability of having kidney cancer throughout one’s lifetime is around 1 in 75 (1.34%) [2]. Renal cancer (RC) is an acute urological disease that affects over 400,000 individuals each year [3,4]. According to the Global Cancer Observatory (GCO), more than 175,000 deaths are due to this disease [5,6]. Renal cell carcinoma (RCC) has the third-highest disease rate after prostate cancer and bladder cancer [7]. It is estimated that RCC is the seventh most frequent cancer in men and the ninth most common cancer in women in the United States, with 48,780 new cases diagnosed and 27,300 new instances of RCC-related death [8]. On radiography, distinguishing between benign kidney tumors and malignant renal cell carcinoma can be challenging [9]. However, the majority of kidney tumors turn out to be cancerous [10]. Renal cell carcinoma (RCC) represents the vast majority of these tumors [11,12]. Clear cell RCC is the most common subtype of renal RCC [13], accounting for approximately 80–90% of all kidney cancers. Overall, the worldwide incidence rate has increased by 2% per year during the last two decades [8]. Kidney tumors are becoming more common, and the disease develops for a long time without causing symptoms. Coincidence may be the reason for detecting more than half of the cases of renal cell carcinoma [14,15]. Importantly, the cause of kidney cancer has not been determined. However, many risk factors, including smoking, obesity, poor diet, substantial alcohol consumption, family history of hypertension, exposure to radiation and chlorinated chemicals, and heredity, are risk factors for getting sick [16]. Radical nephrectomy (RN) and partial nephrectomy (PN) are the current therapeutic options for kidney tumors. The tumor and kidney are removed in RN, but in PN, only the malignancy is removed [17].

**Figure 2:**
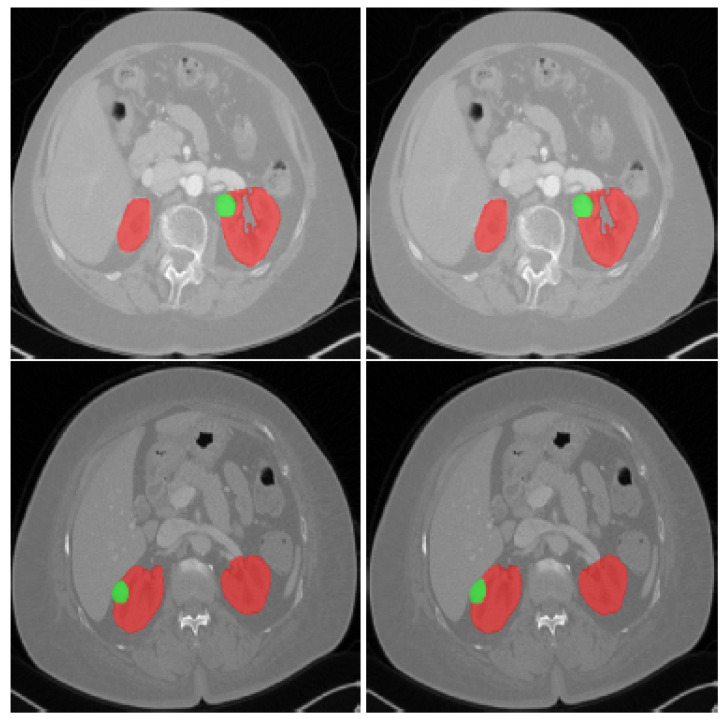
CT scans showing: An axial slice of two patients’ 3D CT scans from the KiTS19 dataset; the red tint denotes the kidneys, whereas the green color indicates the tumor site [5].

Image processing is a widely utilized technology in a range of industries, including robotics, biometrics, security and surveillance, remote sensing, and medical imaging. An image processing task’s overall applicability and performance are heavily dependent on the quality of the test image [18]. Medical imaging techniques are divided into several types: ultrasound sonography (US), computed tomography (CT), and magnetic resonance imaging (MRI). Medical images (MI) have excellent homogeneity, make it challenging to identify regions of interest and patterns and blurring the boundaries between organs and other areas. Radiologists favor CT imaging over other imaging modalities because it produces high-resolution images with good anatomical features. In addition, it gives images with excellent contrast and exceptional spatial resolution. Therefore, CT imaging is an essential tool for diagnosing any disease affecting the kidneys [1]. It is frequently utilized in clinics for therapy planning and segmentation of kidney tumors [19]. In addition to that, some CT results can be utilized to classify benign cancers (Figure 2) [20]. The most common method of tumor delineation is by hand. An expert radiologist will carefully examine the patients scanned medical photographs, segmenting all damaged areas. Manual segmentation is time-consuming. It also has a lot of intra- and inter-rater variability [21]. CT technologies can significantly enhance our ability to detect and monitor diseases and patients, and this may improve patient care and facilitate the evaluation of treatment strategies.

Image segmentation is the process of splitting an image into several segments in order to transform it into a more meaningful and easy-to-analyze representation [22]. The process of image segmentation may be conceived of in two steps: identification and delineation. Identification is the process of identifying the location of an object in an image and differentiating it from everything else in the image. Segmentation involves delineating the boundaries of the region of interest for further analysis [1]. There are several methods for segmenting images: Manual Segmentation, Semi-Automatic, Automatic Segmentation, and Semantic Segmentation. Semantic segmentation is crucial for image analysis tasks and plays a significant part in image interpretation. Image categorization, object recognition, and border localization are all required for semantic segmentation [23]. Semantic segmentation has several applications in computer vision and artificial intelligence-assisted autonomous driving, and medical imaging analysis [24,25,26].

Since scanning and loading (MI) onto a computer became practical, researchers have created automated analysis tools. MI analysis was done between the 1970s and 1990s by combining low-level pixel processing (edge and line detector filters, region expansion) with mathematical modeling (fitting lines, circles, and ellipses) to create compound rule-based systems that handled specific tasks [27]. One of the most challenging problems in medical image analysis (MIA) using traditional approaches such as edge detection filters and mathematical algorithms is distinguishing the pixels of organs or lesions from background medical images of CT or MRI scans to give vital information on the shapes and sizes of these organs. Therefore, researchers have suggested numerous automatic segmentation methods to extract the hand-crafted characteristics, such as machine learning techniques [28]. Around the 1990s, supervised approaches involving training data to develop a system became more prevalent in medical image analysis. Active shape models (for segmentation), atlas techniques (in which atlases are fitted to fresh data extracted from the training data), feature extraction, and statistical classifiers are just a few examples (for computer-aided detection and diagnosis). This pattern recognition or machine learning technique is still frequently utilized, and it is the basis for a large number of commercially accessible medical image analysis products. As a result, we have seen a shift away from human-designed systems toward systems that computers train using example data and extract feature vectors. In the high-dimensional feature space, computer algorithms find the best decision boundary. The extraction of discriminant characteristics from images is a critical step in the construction of such systems. Humans still carry out this procedure, and as a result, one speaks of systems with hand-crafted features [27]. As a result of these technological advancements, deep learning techniques began to exhibit their significant capabilities in image processing applications [28]. DL is a type of machine learning that enables more precise and quicker results than traditional machine learning techniques [29].

Recently, DL techniques for semantic image segmentation have shown promising results in a variety of medical image analysis applications [30,31,32]. Convolutional neural networks (CNNs) are the most successful form of image processing model to date. CNN have multiple layers [27]. CNN has been under development since the late 1970s by Fukushima. Moreover, they were used to analyze medical images in 1995 [33]. They witnessed their first successful real-world application in 1998 for hand-written digit recognition. In the December 2012 ImageNet competition, Alex-Net, the planned CNN, won the competition by a huge majority. Using analogous but deeper designs, more work was done in later years. Deep convolutional networks have emerged as the preferred method for computer vision [27]. Due to their powerful non-linear feature extraction capabilities and the efficiency of encoder–decoder architectures, CNNs have been used for complex segmentation tasks [5]. In computer vision tasks, CNN architectures have already surpassed classical methods [12], particularly in the segmentation of CT scans [34]. On the other hand, fully convolutional network (FCN) architectures are a notably powerful end-to-end training segmentation solution. This architecture is state-of-the-art across several domains and can create raw-scale, pixel-level labels on the images [12]. Other subsequent efforts, such as the feature pyramid networks (FPN), primarily utilized in object recognition, have used FCN as a starting point for deeper and more sophisticated segmentation structures [35]. Pyramid scene parsing networks (PSPNets) are used to analyze scenes [36]. For object instance segmentation, Mark R-CNN [37] is used.

Semantic segmentation using DL techniques: DL, a proliferating new machine learning division, has proven their effectiveness in semantic segmentation. Deep learning techniques play an important role in easing image understanding [23,38]. Deep learning techniques for semantic segmentation have been divided into region-based, FCN, and semi-supervised methods. Region-based methods adopt the pipeline method by first extracting free-form regions from input images, then classifying them using region-based classification; finally, they label pixels according to the scoring regions [38]. FCN-based methods, in contrast to region-based methods, do not extract the region proposal. They learn a mapping from pixel to pixel, making arbitrary-sized images [38]. Regarding semi-supervised methods, usually, semantic methods depend on many images that require a long time to annotate the masks. Therefore, some semi-supervised methods have been suggested to utilize the annotation process [38]. In addition to the methods mentioned, more DL categories for semantic segmentation have been proposed, including feature encoder-based methods, recurrent neural network-based methods, upsampling/deconvolution-based methods, increased resolution of feature-based methods, enhancement of feature-based methods, spatiotemporal-based methods, and methods using CRF/MRF [23].

Semantic segmentation is a hot research area in medical image processing. This is particularly true for abdominal CT scans, where many contests push academics to continue developing approaches for improving segmentation performance [39]. Even though medical segmentation is becoming increasingly prevalent, there are few ways for segmenting kidney and renal tumors in the literature [40]. To our knowledge, only a few review articles have examined kidney segmentation strategies. Nonetheless, numerous articles have been published on the subject of kidney segmentation. Additionally, various deep learning-based algorithms have been developed for segmenting images of other diseases, such as skin lesions, dental imaging, and eye images. As a result, the research barriers for adopting and applying these approaches to the kidney images have encouraged study on kidney segmentation and detection. This study look at cutting-edge DL techniques for segmenting CT kidney cancers. Additionally, the article highlights distinct challenges and possible solutions for medical image interpretation.

## 2. Related Work

In recent years, we have seen tremendous advancement in development in many fields as processing power have increased. Among the technologies that have succeeded and evolved are those that are based on deep learning, most notably CNNs, which have been successful in medical image processing. It has risen to the top of the list for various medical image analysis applications, including medical image segmentation detection, abnormality classification, and medical image retrieval. Many interesting techniques have appeared in the field of medical image segmentation using DL [41]. Moreover, the problem of kidney image segmentation has attracted research earlier. Some approaches, such as atlas-based methods, deformable models, graph cuts technique, and region growing, have been developed and used. In [42] the authors describe the shapes of the local objects via contour templates, which are used to capture specific properties of different organs. The authors in [43] used 2D CNN for the segmentation of computerized tomography (CT) images. At present, end-to-end segmentation and cascade segmentation are the two most commonly used deep learning segmentation strategies in image segmentation. Cascaded segmentation refers to multistage processing to achieve stepwise segmentation, whereas end-to-end segmentation employs only one model to execute the segmentation operation directly. On the other hand, end-to-end segmentation better prevents the buildup of mistakes in multistage segmentation and streamlines the procedure. However, a single model with great integrationreduces its flexibility, operability, and interoperability. Simultaneously, a single model may require additional training data in order to obtain better outcomes. As a result, many researchers still use cascade approaches in medical image segmentation [41].

### 2.1. One-Stage Methods

One-stage methods [39,44,45,46,47,48,49,50,51,52,53,54,55,56] are designed to predict the multi-class results directly from whole images. Myronenko et al. [44], from arterial phase abdominal 3D CT images, presented an end-to-end boundary aware fully CNN for accurate kidney and kidney tumor semantic segmentation. Efremova et al. [45] combined U-Net and LinkNet-34 with ImageNet-trained ResNet-34 to decrease the convergence time and overfitting. Their model has shown success in a wide range of computer vision applications, including medical image processing. Guo et al. [46] proposed an automatic segmentation model called RAU-Net. Their model has been developed for renal tumor segmentation. With some utilization of the cross-entropy function, the model can identify positive samples. However, their generalizability is lacking. Isensee et al. [39] designed a U-Net model that performs well with the KiTS2019 dataset and is able to learn segmentation tasks from reference data. To achieve a regularizing effect, you can either lower the number of layers in the typical U-Net or increase the number of residual blocks. Causey et al. [47] proposed a deep learning model (Arkansas AI-Campus) that collects U-Net models produced after several model variants were tested. Their model performs consistently on both the local test dataset and the final competition independent test dataset; it takes place in the top five of the KiTS19 Competition among US teams. Nazari et al. [48] developed a DL technique to detect the borders of organs with high accuracy using computed tomography images. They used the obtained inches to calculate dosimetry using cautery as the source organ. Yasmeen et al. [49] demonstrate a deep neural network cascaded for semantic segmentation of kidneys and surrounding anatomy. Ruan et al. [50] much of the work focuses on the feature map at the network’s bottom, which enhances network performance by extracting and fusing multi-scale information. Yu et al. [51] it is recommended that non-squared patches with varying aspect ratios be used to train segmentation networks in order to integrate more global contexts in local details. According to Pang et al. [52], automatic image segmentation is a frequent application case in machine learning that has gotten much attention in recent years. Tumor segmentation in computed tomography (CT) images is a popular application. Shen et al. [53], to cope with kidney and tumor segmentation problems, suggested the COTRNet. COTRNet uses a transformer to capture long-range dependencies for accurate tumor segmentation, inspired by the DETR, which used one to represent global characteristics. Yang et al. [54] suggested a 3D fully convolutional network with a pyramid pooling module intended specifically for segmenting kidney and renal pathologies. Experiments and comparisons with other methods show that their method performs very well, with an average dice coefficient of 0.931 for kidney segmentation and 0.802 for tumor segmentation. To make an attempt at resolving the class imbalance, Heo et al. [55] presented a one-stage semantic segmentation model based on 3D abdominal CT imaging for the KiTS21 Challenge. The model was constructed using U-Net and the sum of the Focal and Dice Losses. To improve performance. Christina et al. [56] benefited from a strategic sampling approach based on clinical data. Using random sampling, a baseline 3D U-Net was trained. The clinical features most strongly related to segmentation success were determined using LASSO regression and then included into a mindful sampling method, maximizing the influence of the identified clinical characteristics.

### 2.2. Two-Stage Methods

The goal of the two-stage approach is to overcome the problem of foreground/background imbalance. Those approaches begin by detecting the volume of interest (VOIs) and then segmenting the target organs from the VOIs (VOIs) [57]. Regarding two-stage methods [12,41,58,59,60,61,62,63,64,65,66,67,68,69], Cruz et al. [58] developed a method that uses deep convolutional neural networks with image processing techniques to delimit the kidneys in CT images, where they achieved up to 93.03% accuracy, so further improvements are required. Zhang et al. [59] studied a cascaded two-stage framework using a 3D fully convolutional network (FCN) for kidney and tumor segmentation. Their method locates the kidney and cuts off the irrelevant background. Hou et al. [60] offered a triple-stage self-guided network for the kidney tumor segmentation challenge. From down-sampled CT images, the low-resolution net can approximately find the volume of interest (VOI). Still, the full-resolution net and tumor refine net can extract the right kidney and tumor borders inside VOI from full-resolution CT images. Their model squanders computational resources while training numerous networks. Hatamizadeh et al. [61] enhanced the edge representations in learned feature maps with their module that can be combined with any generic encoder–decoder architecture; the core idea of their model is to add an extra task of learning edges to the original network. Some researchers attempt to push their networks to learn specific characteristics in an ambiguous and multitasking manner, which is unacceptably inefficient. Zhao et al. [12] developed a U-Net-based model called MSS U-Net, a multi-scale supervised 3D U-Net for segmenting kidneys and kidney cancers from CT scans. They combined deep supervision with exponential and logarithmic loss to improve the efficiency of 3D U-Net training. Santini et al. [62] combined Res-Net with Res-U-Net architectures in a multi-stage DL approach called EMS-DLA that has been used for kidney tumor segmentation. The results are promising, and they might be improved if an enhanced understanding of benign cysts is factored in. Xie et al. [41] presented a cascaded SE-ResNeXT U-Net. Chen et al. [63] presented a technique for segmenting kidney, tumor, and cyst in abdomen enhanced CT images based on a multi-stage stepwise refinement strategy. In network training, a 2.5D technique is utilized for data input to maintain certain contextual semantic information while reducing memory strain. There are certain points in this report that need to be further looked at. For smaller kidneys, tumors, and cyst segmentation, the network and procedures can be enhanced. Wei et al. [64], to differentiate kidney cancers, employed two-phase models, which are cascaded network structures. In a tumor, they achieved 0.75.He et al. [65], for kidney segmentation, suggested a novel two-stage cascade and multi-decoding approach. They used U-Net to locate and extract the kidney area and then MSD-Net for final segmentation. Yi Lv et al. [66] offered a three-step automated kidney tumor and cyst segmentation approach based on 3D U-Net. According to the findings, the average dice for kidneys, tumors, and cysts is around 0.93, 0.57, and 0.73, respectively. The accuracy of tumors and cysts, on the other hand, is not sufficient. Li et al. [67] presented a two-stage cascaded segmentation technique for the kidney, renal tumor, and renal cyst. This was accomplished by embedding a Residual 3D U-Net architecture into each level of the cascaded process. The suggested approach demonstrated good segmentation outcomes for the kidney and tumor. Because the border between the tumor and the kidney is ambiguous, the segmentation of the kidney and tumor is complicated. Xiao et al. [68] suggested a two-stage detection and segmentation architecture for autonomously segmenting kidneys, cysts, and tumor based on the KiTS21 benchmark. The ResUnet 3D was used as the foundation. The two-stage architecture produced a mean dice of 0.6543 for kidney and messes, 0.6543 for kidney messes and tumor, and 0.4658 for the mean surface dice. Wen et al. [69] They presented a unique segmentation network named SeResUNet to segment the kidney and tumor. Here, one must choose an encoder–decoder architecture such as U-Net and utilize ResNet to deepen the encoder’s network and, simultaneously, to minimize severe network degradation and accelerate convergence.

### 2.3. Hybrid Models

Tow introduced hybrid models in [70,71,72]. Abdul Qayyuma et al. [70] designed a hybrid 3D residual network (RN) with a squeeze-and-excitation (’SE’) block to acquire spatial information by utilizing cSE blocks. The reweighting function in a “three-D RN” is used. Their network has been tested on various datasets and performs well in medical image segmentation, especially in volumetric segmentation of the kidney, liver, and related malignancies. Cheng et al. [71] enhanced 3D SEAU-Net to develop a multi-class segmentation architecture to improve the performance. Their model aggregates residual network, dilated convolution, squeeze-and-excitation network, and attention mechanism. The multi-class segmentation job is decomposed into two more easy binary segmentations. Cruz et al. [72] provide an effective approach for segmenting kidney cancers in computed tomography. Thus, post-processing was applied on the DeepLabv3+ 2.5D model with DPN-131 encoder. Additionally, image processing methods such as normalization, proportional distribution of training and validation datasets, and DART were employed. Finally, the findings were achieved by integrating preprocessing, segmentation of the kidney tumor, and postprocessing.

## 3. Overview of Deep Learning (DL) Models

**Figure 3:**
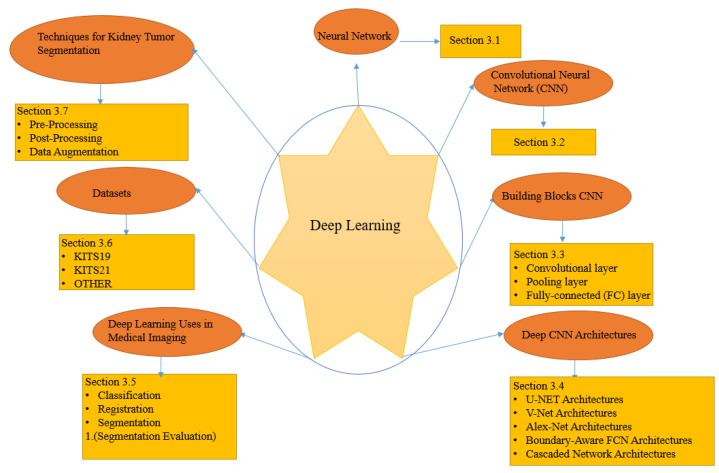
Components architectures, and strategies for deep learning algorithms for segmenting kidney tumors.

This section discusses the DL concepts, techniques, and architectures of DL algorithms for kidney and renal tumor segmentation that we discovered when reviewing the medical image analysis articles examined in this work, as summarized in Figure 3.

DL is an artificial intelligence (AI) function concerned with decision-making algorithms inspired by the structure and function of the human brain, referred to as “artificial neural networks”. It is a branch of machine learning in artificial intelligence that utilizes networks capable of unsupervised learning from unstructured or unlabeled input. They are also referred to as deep neural networks or deep neural learning. In a variety of medical image analysis applications, semantic image segmentation algorithms based on deep learning have demonstrated promising results. Deep CNNs have been state-of-the-art for many image classification and segmentation applications. CNNs have been used for complex segmentation problems due to their superior non-linear feature extraction capabilities and the efficacy of their encoder–decoder architectures [73].

### 3.1. Neural Networks

**Figure 4:**
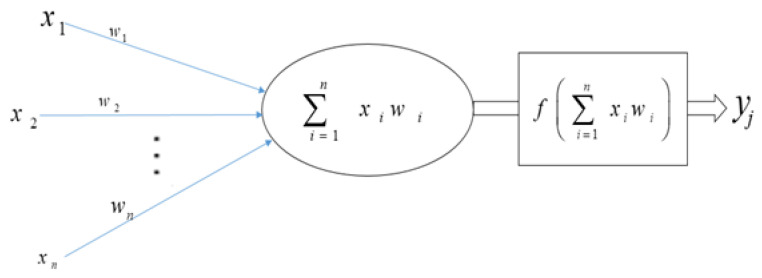
The building block of deep neural networks is an artificial neuron or node.

Neural networks are a sort of learning algorithm that serves as the foundation for the majority of DL techniques [21,27]. As seen in Figure 4, neural networks are nothing more than a set of arithmetic operations that convert an input (x) into an output (y). Weights and linear operators multiply the previous value of connected input and output to the neuron. The function in the hidden layer can be anything. In the simplest example, with a single neuron in the hidden layer (A), the input value is multiplied by the first weight, and the resulting value Xxweight1 is then passed to the neuron’s function. The result is calculated by multiplying the output of that function by weight2. When a neural network is trained, it is fed inputs and the outcome is computed using mathematics. The output value is compared to the known real value of y; the weights are slightly modified to get the output value closer to the known actual value of y. A straightforward illustration of this is presented in (B), where weight1 equals 2, function equals 2Xx, and weight2 equals 2 [74].

**Figure 5:**
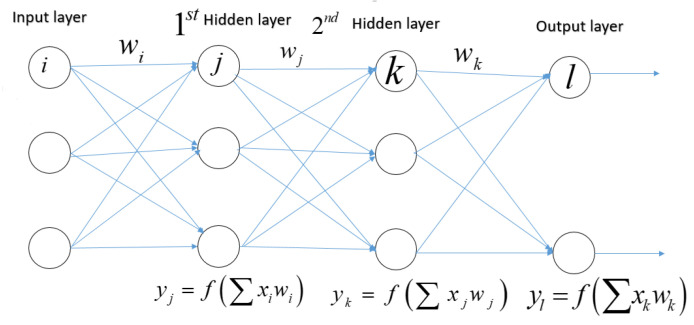
Artificial feedforward multilayer neural network.

Each input $x_{i}$ has a corresponding weight $w_{i}$ in an artificial neuron or node.The total of all weighted inputs ($x_{i},w_{i}$) is then fed into a nonlinear activation function (*f*), which translates the preactivation level of the neuron to an output ($y_{j}$).The bias terms have been deleted for simplicity. The result (y)j) is then used as an input for the following layer’s node. Numerous activation functions are available, each with a somewhat different mapping of a preactivation level to an output value. The rectifier function is the most often activated function (neurons that employ it are referred to as “rectified linear units” (ReLU)), followed by the hyperbolic tangent function, the sigmoid function, and the softmax function. As seen in Figure 5, the latter is frequently utilized in the output layer to compute the likelihood of multiclass labels.

A feedforward multilayer neural network with two classes (also known as a multilayer perception). Each node in one layer is linked to every neuron in the subsequent layer (a fully connected network). The weighted total of the inputs for each neuron j in the first hidden layer is multiplied by a nonlinear function. The output ($y_{j}$) of this transformation is utilized as the input for the second hidden layer. The information is transferred to the output layer through the network.

### 3.2. Convolutional Neural Network (CNN)

**Figure 6:**
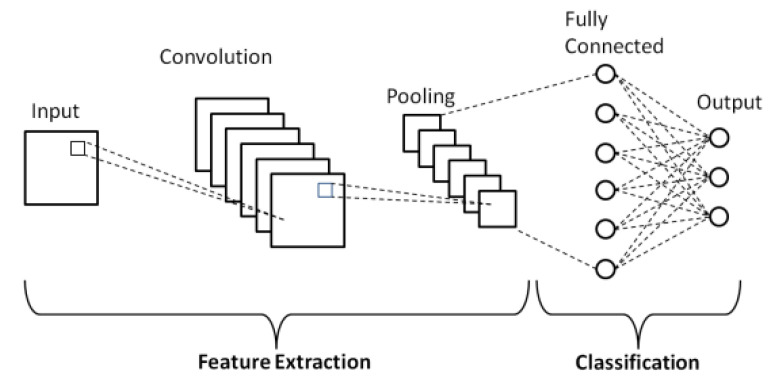
Schematic diagram of a basic convolutional neural network (CNN) architecture [19].

A CNN is a kind of neural network. It was first released in 1980 [75]. CNNs have established themselves as the de facto technique for handling a wide variety of challenging computer vision issues in recent years [76]. CNN is one of the most recent DL algorithms in image recognition. CNN is inspired by the multi-layered structure of the visual cortex in the human brain and has demonstrated outstanding performance in a variety of very complicated application scenarios [76,77]. The traditional machine learning technique to image identification consists of two distinct processes. Using several techniques, such as HOG [78], SURF [79], or HOUP [80], the initial stage, dubbed feature engineering, attempts to extract meaningful data representations from the raw image data. In the second stage, referred known as classification, a machine learning algorithm attempts to discover a pattern that links previously created data representations to a target variable. The algorithm is only capable of learning these patterns if they have already been retrieved via feature engineering. Manual extraction of appropriate data representations, in particular, frequently results in unsatisfactory categorization results [76]. The combination of these two phases is the essential distinction between convolutional neural networks and standard machine learning algorithms for computer vision [76]. CNN are feedforward neural networks intended to analyze images and are physiologically inspired by the visual cortex [81]. Numerous convolutional layers are used in common CNN topologies. Each image is processed in three dimensions using a three-dimensional array. By applying several tiny filter kernels to the image array, the convolutional layers convert the original input to feature maps [76], as seen in Figure 6. The filter matrices are applied to the entire image, preserving spatial information. Following that, these feature maps are processed via a nonlinearity function such as ReLU, a batch-normalization layer, a convolutional layer, and a pooling layer. CNN are capable of automatically extracting useful feature representations with fully connected layers from raw images and optimizing them to represent specific target classes by combining several convolutional, activation, batch normalization, and pooling layers [76]. In comparison to alternative models, CNN has recently become the de facto model for medical image segmentation due to its record-breaking performance in conventional computer vision tasks as well as medical image analysis [21]. CNN models may learn spatial hierarchies of features within data, e.g., the first layer will learn tiny local patterns, such as edges, while the second convolutional layer may learn bigger patterns constructed from the first layer characteristics, and so forth. They are more suited to image analysis jobs because of this capacity. Furthermore, convolutional layers’ units share weights, decreasing the number of parameters to train and improve the network’s efficiency [21]. CNN has recently become the de facto paradigm for kidney tumor segmentation because of its superior performance in traditional computer vision and medical image analysis when compared to alternative models. CNN models can learn to create spatial hierarchies of features included inside data.

A CNN’s architecture is hierarchical. Starting with the input signal, each succeeding layer $x_{j}$ is calculated as follows [82]:

$$x_{j}=PW_{J}X_{J}-1$$

Here $W_{J}$ is a linear operator, and ρ is a non-linearity. Typically, in a CNN, $W_{J}$ is a convolution, and ρ is a rectifier max(x,0) or sigmoid $\frac{1}{1+exp(-x)}$. It is easier to think of the Operator $W_{J}$ as a stack of convolutional filters. Thus, the layers are filter maps and each the layer can be expressed as the sum of the previous layer’s convolutions [82].

$$x_{j}\left(u,k_{j}\right)=\rho (\sum_{k}\left(x_{j}-1\left(.,k\right)*W_{j},k_{j}\left(.,k\right)\right)\left(u\right))$$

Here is the discrete convolution operator:

$$\left(f*g\right)\left(x\right)=\sum_{u=-\infty }^{\infty }f\left(u\right)g\left(x-u\right)$$

A CNN’s optimization issue is substantially non-convex. Thus, the weights $W_{J}$ are often taught by stochastic gradient descent, with the gradients computed using the backpropagation process [82].

### 3.3. Building Blocks CNN

A convolutional network’s initial layer is the convolutional layer. Convolutional layers can be followed by additional or pooling levels, but the wholly linked layer is the final layer. The CNN becomes more sophisticated with each layer, recognizing larger areas of the image. Earlier layers concentrate on essential elements like colors and borders. As the image data goes through the CNN layers, and it detects more significant components or forms of the item, eventually identifying the desired object. They have three main types of layers, which are:

- Convolutional layer.
- Pooling layer.
- Fully-connected (FC) layer.

#### 3.3.1. Convolutional Layer

This layer will determine the output of neurons connected to local regions of the input through the calculation of the scalar product between their weights and the region connected to the input volume. This layer is the central constituent of a CNN, and it is here that the majority of the processing takes place. It requires input data, a filter, and a feature map, among other things. This layer consists of a set of learnable filters or kernels (the typical size is usually 3 × 3 or 3 × 3 × 3, depending on whether the input is a two-dimensional (2D) or three-dimensional (3D) image, respectively) [21].

#### 3.3.2. Pooling Layer

A pooling layer is typically used in conjunction with a convolutional layer or a collection of convolutional layers. The objective is to minimize the size of the feature maps while retaining critical features. A sliding window pooling method is conducted to a rectangular neighborhood. For instance, max pooling is used to maximize the size of a rectangle neighborhood. Additionally, average and weighted average pooling are prominent pooling methods [21]. Similar to the first layer, the pooling procedure sweeps a filter across the whole input, except this filter does not include any weights. Rather than that, the kernel populates the output array with values from the receptive field using an aggregation function. Pooling may be classified into two types: Max pooling, Average pooling. Pooling is primarily concerned with down sampling in order to lessen the complexity of subsequent layers. In the sphere of image processing, this is analogous to decreasing the resolution. The number of filters does not change as a result of pooling. Max-pooling is one of the most often used pooling techniques. It divides the image into rectangular sub-regions and returns just the largest value contained inside each sub-region. One of the most often utilized sizes in max-pooling is 2 × 2 [82].

#### 3.3.3. Fully Connected (FC) Layer

The moniker “fully connected layer” is self-explanatory. As previously stated, with partially linked layers, the pixel values of the input image are not directly connected to the output layer. By contrast, each node in the output layer connects directly to the previous layer in the eventually linked layer.This layer performs classification tasks based on the characteristics collected by the preceding layers and their various filters. While convolutional and pooling layers often employ ReLu functions to categorize inputs, (CL) are used to extract features. The characteristics they generate are subsequently classified by the fully connected (FC) layers. As seen in Figure 6, each unit in the FC layer is connected to every unit in the preceding layer. Typically, the last layer is a softmax classifier that generates a probability vector map across the various classes. Prior to passing the features to an FC layer, they are all transformed to a one-dimensional feature vector. This results in the loss of spatial information included in image data. A disadvantage of the FC layers is that they have a greater number of parameters than other layers, raising computing costs and needing identical input images. The primary disadvantage of a fully-connected layer is that it has a large number of parameters that need complicated calculation during training. As a result, we attempt to reduce the number of nodes and connections. The dropout approach can be used to satisfy the deleted nodes and connections [21,82].

### 3.4. Deep CNN Architectures

Given the widespread usage of CNNs in medical image processing, we discuss common designs and architectural distinctions across the most extensively used models.

#### 3.4.1. U-NET Architectures

U-Net is a CNN developed for biomedical image segmentation at the Computer Science Department of the University of Freiburg [83]. The network is based on convolutional networks. The encoder–decoder design of U-Net is a stable network for medical image segmentation. Moreover, its architecture was modified and extended to work with fewer training images and more precise segmentation. Segmentation of a 512 × 512 image takes less than a second on a modern GPU [83]. Ronneberger et al. [83] developed a U-Net network which consists of two stages (path contracting and symmetric expanding). Path contracting is used to capture, while symmetric expanding is used for precise localization. However, the U-Net architecture depends highly on data augmentation techniques. The architecture of U-Net is composed of 3 × 3 convolutional layers. Maximum of 2 × 2 follows each pooling layer and the ReLU activation function.Finally, the 1 × 1 convolutional layer is attached. The encoder–decoder design of U-Net [83] is a sustainably successful network for medical image segmentation. Because volumetric data is more abundant than 2D images in biomedical data analysis, 3D convolution is suggested and considered considerably more successful in fully using the spatial information of 3D images such as CT and MRI. Based on 3D U-Net [83], Fabian [84] made minor changes. He placed first in several medical image segmentation contests, demonstrating that an optimized U-Net can outperform many other innovative designs.

#### 3.4.2. V-Net Architectures

This section refers to the the proposed V-Net structure in [32]. As with the fundamental U-Net design, the network architecture of the V-Net is composed of encoding and decoding components. Thus, it is a derivation of the U-Net architecture, but with a volumetric design that makes it suited for usage in tissues where organs and tumors are difficult to recognize on CT imaging (such as the prostate or kidney) [32]. The convolutional nature of the V-Net architecture enables it to extract features and reduce the resolution by following the correct route. Because conventional pooling approaches sometimes overlook critical features during the segmentation process, V-Net convolutions are employed to overcome this by downsampling the data provided as input and transmitting it to the receiving characteristics derived in the subsequent network layers [40]. Unlike standard neural network architectures for classification, completely convolutional networks [85], along with the U-Net and the V-Net, do not use a flattening process or contain fully connected layers. Rather than that, it employs upsampling techniques that enable the network to produce an image of the same size as the input, which may address segmentation issues. The V-Net structure is composed of two sections: one for downsampling and one for upsampling. All pooling layers created during the downsampling phase are converted to up-convolution layers during the upsampling phase. Additionally, a “contracting path”, as they termed it, is constructed at each layer from the up-sampling to the down-sampling portion in order to concatenate the data. This method enables the network to view high-resolution data again during the up-sampling phase [86]. Each layer on the encoder side of the V-Net architecture is composed of two times the number of feature set computation sections as the preceding layer. The network’s decoder portion is designed to perform two-channel volumetric segmentation. As a result, feature maps are included to assist in obtaining the relevant information. Following each layer in the encoder portion of the network design, a deflection operation is done to increase the size of the entries, followed by the opposite action in the decoder section to decrease the dimensions. The encoder phase’s neural network attributes are passed to the decoder phase. This is schematically depicted by the use of horizontal linkages [83].

#### 3.4.3. Alex-Net Architectures

CNN is available in many different configurations, including Le-Net, Alex-Net, Google-Net, Conv-Net, and Res-Net. Additionally, we chose the Alex-Net design since it is more resilient to difficulties than alternative designs. Alex-Net has an eight-layer architecture, with the first five layers being convolutional and maximum pooling layers, and the latter three being wholly linked to the neural network [87].

#### 3.4.4. Boundary-Aware FCN Architectures

Although U-Net overcomes the challenge of preserving the original information during FCN upsampling via skip-connection, the boundary cannot achieve a reasonable segmentation result due to the blurring of the border and internal pixels. This uncertainty arises because convolution operators might provide comparable values in the voxel feature map at the tumor boundary, even in the first convolution layer [88]. Shen et al. [89] proposed a boundary segmentation technique based on a Boundary-Aware FCN network. The Boundary-Aware FCN transforms the single network segmentation problem into a multitasking network. The two FCN networks share the downsampling phase but operate independently of one another during the upsampling phase. The two upsampling correspond to distinct segmentation goals; the first segments the tumor territory, while the second segments the tumor border. The two segmentation results are then fused, and the final segmentation result is produced after numerous convolutional layers.

#### 3.4.5. Cascaded Network Architectures

In a cascaded architecture, the outputs of each CNN are concatenated [90]. The output of one CNN becomes a direct input to another in this architecture. The input cascade is utilized to provide extra image channels to the second CNN by concatenating contextual information. This is an improvement over the dual-path approach, which separates multi-scale label prediction from multi-scale label prediction. Concatenation of local pathways is another variant of cascaded design [90]. Instead of concatenating the output of the first CNN with the output of the second CNN’s first hidden layer, this architecture concatenates the output of the first CNN with the output of the second CNN’s first hidden layer. Segmentation in a hierarchical fashion [91]: Tumor segmentation is related to organ segmentation in that tumor segmentation needs the location of the tumor based on organ segmentation. A cascading network is proposed as a result of this. The first neural network determines the organ in which the lesion is located, a process termed “rough segmentation”, and the second neural network determines the precise tumor segmentation. As for the two-stage cascaded tumor segmentation network, the entire network topology is composed of two U-Net networks. To begin, the original image is sent over the first U-Net network, which converts it to a binary image. After multiplying the image by the original, it is delivered to the second U-Net network. The output result is the segmentation result in its entirety [88].

### 3.5. Deep Learning Uses in Medical Imaging

#### 3.5.1. Classification

Classification of images or exams was one of the first areas where DL significantly contributed to medical image analysis. Typically, in exam classification, one or more photographs (an exam) are used as input, and a single diagnostic variable is used as an output (e.g., disease present or not). Each diagnostic assessment serves as a sample in this environment, and dataset sizes are often less than those used in computer vision (e.g., hundreds/thousands of samples vs. millions of samples). Transfer learning’s appeal for such applications is unsurprising. Transfer learning is the process of utilizing pre-trained networks (usually on natural imagery) in order to circumvent the (perceived) demand for big data sets for deep network training. Two ways of transfer learning were identified: (1) employing a pre-trained network as a feature extractor, and (2) fine-tuning a pre-trained network using medical data. The former technique also has the advantage of avoiding the need to train a deep network, which enables the extracted features to be simply integrated into existing image analysis pipelines. Both tactics are frequently used and popular [27].

#### 3.5.2. Registration

Registration (or spatial alignment) of medical images is a common image analysis task that entails computing the coordinate transform between two medical images. This is frequently done iteratively, with a specified (non-)parametric transformation assumed and a predefined metric (e.g., the L2-norm) optimized. While segmentation and lesion detection are more important topics in deep learning, researchers have demonstrated that deep networks can assist in reaching the highest possible registration performance. In general, two techniques are prevalent in the current literature: (1) employing deep learning networks to quantify the similarity between two images in order to drive an iterative optimization strategy, and (2) directly predicting transformation parameters using deep regression networks [27].

#### 3.5.3. Segmentation

Segmentation is a typical job in both natural and medical image analysis, and CNNs can easily identify each pixel in the image independently by providing it with patches extracted from surrounding the pixel. A disadvantage of this naive sliding window technique is that input patches from adjacent pixels overlap significantly, resulting in several computations of the same convolution. Because both convolution and dot products are linear operations, and inner products may be represented as convolutions and vice versa. The CNN can process bigger input images than those used for training and generate a probability map rather than a single pixel output by rewriting the fully linked layers as convolutions. The resultant “fully convolutional network” (fCNN) may be applied efficiently to an entire input image or volume [27].

On the other hand, pooling layers may result in output with a far lower resolution than the input. Shift-and-stitch [27] is one of the proposed ways to prevent this loss of resolution. On shifted versions of the input image, the fCNN is applied. A full-resolution version of the final output is obtained by sewing the result together, minus the pixels lost due to the “valid” convolutions. Ronneberger et al. [83] undertook the fCNN concept a step further and suggested the U-Net architecture, which consists of a regular fCNN followed by an upsampling section where up-convolutions are utilized to expand the image size. He invented the terms “contractive” and “expansive” routes. Milletari et al. [32], instead of the standard cross-entropy, presented a modification to the U-Net architecture that integrates ResNet-like residual blocks and a Dice loss layer that directly reduces this extensively used segmentation error metric.

#### 3.5.4. Segmentation Evaluation

Image segmentation has a wide range of applications in most areas of digital image processing. As a result, segmentation evaluation will be crucial in a wide range of disciplines [92]. Manual segmentation defines the boundaries of the Region of Interest (ROI). The bulk of studies in the literature employ manual segmentation to estimate renal volume [93,94]. To check if the segmentation method is valid, first establish the true boundaries of the region of interest. Unfortunately, the major difficulty with medical image segmentation approaches are the lack ground truth. Manually segmenting the Region of Interest (ROI) from the image and comparing those ROIs to ROIs generated by the segmentation method regarding boundary differences or overlap, is a typical solution to this problem [1]. The accuracy of the proposed kidney segmentation method is assessed by comparing the ground truth marked by professional radiologists with the output of the recommended algorithm. Measures for evaluation may be divided into two categories: quantitative and qualitative metrics. A quantitative review includes obtaining mathematical values, whereas a qualitative evaluation requires visually comparing the ground reality with the silhouette generated [1]. The ground truth and the results are compared using error measures. The following are some of the metrics that have been utilized in the literature: Dice Similarity Coefficient (DSC), Specificity (SP), Sensitivity (SN), Accuracy (AC), Jaccard index (JI), Hausdorff Distance (HD), Area Overlap (AO), Area Overlap Error (AOE), Surface Distance (SD), Volume Overlap (VO), Relative Volume Difference (RVD), and Volume Overlap Error (VOE) [95,96,97]. Some of the various metrics used in the literature are presented in Table 1.

### 3.6. Datasets

**Figure 7:**
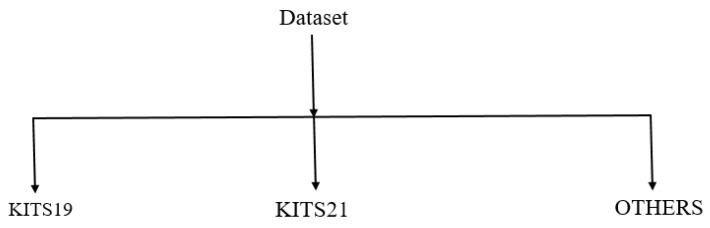
Type of dataset in papers surveyed.

There has been substantial scientific interest in automated kidney and renal tumor segmentation during the last few years. As research output increased, objective assessment of various algorithms got more difficult due to researchers’ usage of private datasets with various features. Figure 7 and Table 2 summarize the most frequently used datasets for segmenting kidney tumors.

The KiTS19 and KiTs21 Challenges and additional datasets provide a sizable dataset that enables a variety of segmentation tasks. The challenge intends to further research on general-purpose segmentation algorithms capable of performing a variety of tasks without the need for human assistance for kidney segmentation and tumors.

### 3.7. Techniques for Kidney Tumor Segmentation

#### 3.7.1. Pre-Processing

Typically, data is preprocessed before to being transmitted to the deep learning network. Preprocessing is grouped into four categories: approach based on image attributes, data augmentations, noise removal, and edge improvement. They have essentially agreed that if the image is placed directly into the deep neural network without preprocessing, the effect is considerably reduced, and in some cases, proper preprocessing is critical to the model’s performance [88,104]. Among the different bias field correction strategies are the non-parametric non-uniform normalization (N3) approach [105]. It has emerged as the preferred methodology owing to its ease of use and availability as an open-source project [106]. This approach was further enhanced in [106], and it is now often referred to as N4ITK. These approaches are intended to be used with a single image. As a result, the intensity normalization provided by Nyul et al. [107] may be used to achieve a consistent intensity distribution across patients and acquisitions. Another often used preprocessing approach is normalizing the image dataset such that it has a mean of zero and a standard deviation of one. This approach aids in the de-biasing of characteristics. Cropping an image may also be used to eliminate as many background pixels as feasible.

#### 3.7.2. Post-Processing

The output of the deep neural network may be used directly as the result of tumor segmentation. However, the output of a deep neural network is not always immediately applicable to the demands and is not interpretable. As a result, some researchers perform post-processing techniques on the output of DL to obtain more accurate findings. Post-processing is used to fine-tune the segmentation findings. It aids in reducing the number of misclassifications or false positives in segmentation results when using algorithms such as conditional random fields (CRF) [108] and Markov random fields [32]. CRF and MRF-based techniques effectively eliminate false positives by combining model predictions with low-level image information, such as local interactions of pixels and edges, when performing finer adjustments. These approaches, however, are computationally intensive [109]. Related component analysis entails locating and identifying connected components and removing unnecessary blobs using a simple thresholding approach. Another strategy for reducing false positives around the segmentation image’s boundaries is to perform successive morphological operations such as erosion and dilation.

#### 3.7.3. Data Augmentation

The purpose of data augmentation, which is to increase the number of training sets, is to expand the size of the data set by graphical modification, hence making the model more robust and less prone to overt error. Flip, Rotation, Shift, Shear, Zoom, Brightness, and Elastic distortion are all common strategies for increasing the number of datasets [110]. To emphasize that when dealing with a limited data collection, the advantage of data augmentation is equivalent to the benefit of model update [88]. However, it is easy to implement. The problem of lack of access to huge data in kidney and kidney tumors can be solved using Data Augmentation.

## 4. Overview of Kidney Tumor Semantic Segmentation

This section provides a quick overview of kidney tumor segmentation. Renal segmentation is a complex operation; the difficulty of segmentation varies depending on the imaging modality. Various imaging techniques are available.

### 4.1. Renal Imaging

The size of the kidney was established in the past by X-rays or using renal length for urography. The results obtained using these methods showed several problems. Ultrasound sonography (US), computed tomography (CT), and magnetic resonance imaging (MRI) are examples of photography methods that may be used to examine the size and function of the kidneys. It is the first method (US) for measuring two-dimensional kidney volumes. To obtain 3D data, they employed CT and MRI techniques. Each of the current imaging modalities has its imaging capabilities and may be used in various ways depending on the treatment objective [111]. US can identify cysts, stones, and tumors, among other benefits; it gives good anatomical information without exposing the patient to radiation and enabling a low-cost, real-time inspection. However, the images on US are of poor quality [111]. This is a flaw that makes the segmentation procedure difficult. On the other hand, computed tomography (CT) is a technique that allows for higher-quality imaging and identifies small lesions and cysts. However, there is a drawback to ionizing radiation exposure. The last approach (MRI) is insufficient. Overall, the benefit of MRI is that it provides excellent spatial resolution while posing minimal risk to the patient. Its downside is that it is more expensive [111].

### 4.2. Image Segmentation

What exactly is an image? An image is a mathematical representation of what we can see. An image can be defined as a 2D function like a CT image denoted by f(x,y), where the value or amplitude off at spatial coordinates (x,y) gives the intensity (brightness) of the image at that point. A pixel is a visual element that is represented by each end in an image. The function *f* can also be viewed here:

1. The size of the image array N×X,A where
2. *S* and *M* represent the number of rows and columns.

Thus,

(1) $$A=f\left(x,y\right)=\left(\begin{array}{cccc} f_{\left(0,0\right)} & f_{\left(0,1\right)} & \cdots & f_{\left(0,M-1\right)}\\ f_{\left(1,0\right)} & \vdots & \cdots & f_{\left(1,M-1\right)}\\ \vdots & \vdots & \cdots & \vdots \\ f_{\left(N-1,1\right)} & f_{\left(N-1,1\right)} & \cdots & f_{\left(N-1,M-1\right)} \end{array}\right)$$

Image segmentation, defining objects in images, is the most important operation performed on acquired images. Segmentation, ironically, is required for segmentation, as object knowledge helps with segmentation. In image processing and computer vision, segmentation is still a difficult task [112]. The assessment of segmentation algorithms is a closely connected and strongly interrelated topics. The absence of a uniform framework for evaluating segmentation algorithms is one of the challenges in creating them [112]. The initial stage in creating a wholly automated perception system is image segmentation. It has been regarded as a primary challenge in computer vision. Generally speaking, the mission of segmenting an image into parts might be to identify objects or districts of interest [113]. The following section explains the types of image segmentation:

### 4.3. Types of Segmentation

The degree of human participation in image segmentation algorithms may be classified into four types: manual segmentation, semi-automatic segmentation, fully automatic segmentation, and semantic segmentation [114]. A summary of the advantages and disadvantages of segmentation techniques is given in Table 3.

#### 4.3.1. Manual Segmentation

Manual segmentation is done drawing the member or sketching the member’s borders, manually executed by a professional (physician, trained, technician, etc.) [115]. The person operating the machine uses specialized tools to draw or paint around tumor regions. Manual segmentation is a time-consuming and tiresome process. The result of segmentation depends on the training and experience of the person. It also includes what is called “inter-rater” variability (the image is segmented differently by various specialists) and “intra-rater” variability (at various times, the same person segments the image differently). It serves as the basis for various segmentation algorithms (semi-automatic, automatic, and semantic). Even though manual segmentation is dependent on the rater, it is still commonly utilized in clinical studies [116,117,118].

#### 4.3.2. Semi-Automatic Segmentation/Interactive Segmentation

To address manual segmentation problems, semi-automated or automatic segmentation approaches have been developed. Semi-automated segmentation approaches necessitate the operator’s one-time manual startup and manual modification of the computer segmentation result [1]. Semi-Automated Segmentation (SAS) takes less time than manual segmentation, but its outcomes are still reliant on the operator [40,114,115].

#### 4.3.3. Fully Automatic Segmentation

To have as little human interaction as possible, automatic segmentation methods involve the segmentation of kidneys from CT images without human intervention, making them free from human errors and biases [1]. Utilizing anatomical knowledge such as volume, form, and position in automated segmentation algorithms is critical for creating a robust algorithm [40,114]. Radiologists like this sort of approach since it is error-free and operator-independent. Several types of segmentation approach in the literature may be classified as thresholding-based methods, region-based methods, model-based methods, atlas-based methods, and hybrid methods depending on the strategy used to segment the region of interest. Depending on the user’s needs, these segmentation approaches can be automated or semi-automatic [1].

#### 4.3.4. Semantic Segmentation

Semantic segmentation, also known as pixel-level classification, aims to group portions of images corresponding to the same object class. This sort of algorithm may be used to recognize road signs, cancers, and medical tools in surgeries, among other things [40]. Semantic segmentation is a particular job that attempts to split an image into semantically meaningful pieces, making it a step farther than image segmentation. (It is important to remember that semantics is a discipline of linguistics concerned with meaning) [119]. It is a high-level task that paves the way for complete scene comprehension in the broad image. The importance of scene understanding as a crucial computer vision problem is underscored by the fact that an increasing number of applications rely on inferring knowledge from images. Classification of an image refers to assigning it to one of the same categories. Detection is the process of locating and recognizing objects. Because it classifies each pixel into its category, image segmentation may be considered pixel-level prediction. In addition, there is a job called instance segmentation that combines detection and segmentation [120].

#### 4.3.5. Semantic Segmentation Metrics

Semantic segmentation metrics play an essential role in evaluating performance techniques. Different semantic segmentation assessment criteria may produce disparate results because it is unclear how to define successful performance segmentation. Pixel accuracy, mean intersection over union, and representing per-class accuracy are three of the most frequently used measures [121].

For all of them, let $n_{i,j}$ be the number of class, pixels predicted to belong to the classes, *j* In addition, let $k_{j}$ = $\sum_{i}$$n_{i,j}$ be the total pixel number belonging to class *i*. If we assume to have a *T* total number of classes, then:

- Pixel accuracy can be computed as:
  $$acc=\frac{\sum_{j}n_{i,j}}{\sum_{i}n_{i,j}}$$
- Mean intersection over union can be computed as:
  $$miou=\frac{1}{T}\sum_{i}\frac{n_{i,j}}{\left(k_{i}+\sum_{j}n_{j,i}-n_{i,j}\right)}$$
- Mean per class accuracy can be computed as:
  $$miou=\frac{1}{T}\frac{\sum_{i}n_{i,j}}{\sum_{i}k_{i}}$$

## 5. Discussion

Deep learning algorithms for medical image processing have garnered considerable attention in recent years. This is reflected in the year-over-year growth in the number of published works [122]. Recent years have seen the use of LD approaches to a wide variety of problems [123], most notably in computer vision [124], natural language processing [125], and computational audio analysis [126]. DL algorithms have outperformed past state-of-the-art performance in a number of these applications. The domain-independent concept of effectively accomplishing high-level tasks via hierarchical layers of learned abstraction [123]. For instance, DL algorithms can be trained on a sufficiently big dataset to segment TC images to segment kidney tumors. As a result of their effectiveness in tackling numerous problems in computer vision, speech recognition, and natural language processing, CNN-based models have been widely utilized in medical image analysis. Table 4 summarizes the deep learning approaches discussed in this paper. Numerous techniques have significant differences in terms of architectural design, with current works following the U-Net [83]. Additionally, numerous approaches have been developed to solve the inherent difficulties associated with semantic renal TC analysis. Table 5 gives an overview of Deep Learning methods for kidney tumor segmentation on other architecture.

### 5.1. Kidney Tumor

While the techniques listed below are quite effective in segmenting kidney tumors, none of them quantifies kidney tumors. CT scans are frequently used to diagnose kidney malignancies, and deep learning-based segmentation of kidney tumors is also focused on CT images. Yang et al. [54] made one of the earliest attempts to use CNNs for this goal. An enhanced residual fully connected network with a pyramidal pool module is presented to segment kidneys and kidney cancers in CT angiography images. To begin, an atlas-based approach is used to extract two zones of interest from the entire images. Each area is home to a single kidney. These patches are fed into a 3D CNN that is profoundly linked, and additional post-processing is conducted using conditional random fields. Efremova et al. [45] and Shen et al. [135] applied U-Net and 3D U-Net to the job of kidney tumor segmentation, with all techniques achieving much better results than conventional methods [51]. The next step was to analyze images from two distinct viewpoints and then link them via two connected networks. Crossbar-Net, a new architecture for automatic segmentation of kidney tumors in CT images, was described. The axial slice was created by stacking three rectangular patches. These patch designs included extra spatial information and were used to train two separate networks with cascading outputs. Cascaded network architectures were used by Yang et al. [54], Vu et al. [136], Lv et al. [137], Mu et al. [19], and Wei et al. [64] to discriminate between kidney cancers. The difference between the two methods is that Yang et al. [54] used a Gaussian pyramid to expand the receptive field in the first stage’s network structure, while Vu et al. [136] increased the number of layers in the cascade network to three, with the first layer obtaining the results directly, the second layer obtaining the tumor and kidney regions, the third layer obtaining the tumor segmentation results using the input of the second layer cascade, and the fourth layer cascading the final results. The two cascade frameworks in [137] are U-Net and V-Net to achieve distinct cascade characteristics. Recently, Xia et al. [138] suggested a two-stage segmentation strategy for the kidney and the space-occupying lesion region. This approach retrieves images using Spatial CNN (SCNN) and Resent, and smooths and matches pixels using SIFT-flow and Madras Rubber Factory (MRF). Table 5 shows an overview of Deep Learning methods for kidney tumor segmentation on other architectures. Table 6 shows a summary of results on KiTs 2019, KiTs2021, and another dataset.

**Figure 8:**
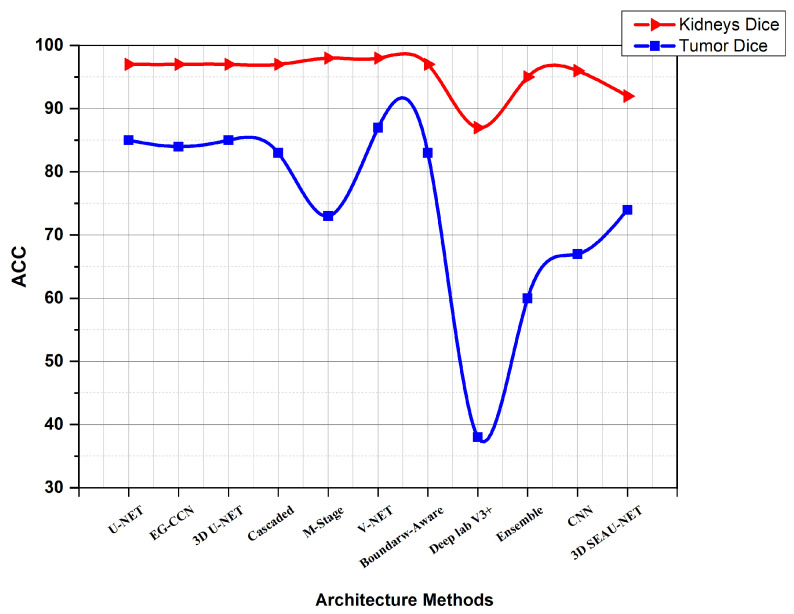
Diagram showing a comparison between different Architecture Methods using KiTs19.

**Figure 9:**
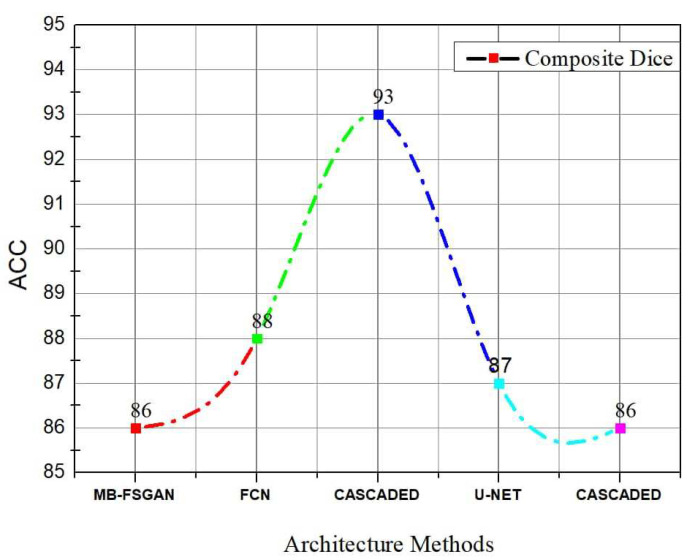
Diagram showing a comparison between different Architecture Methods using KiTs21 and another dataset.

While the method outlined above is quite effective in segmenting kidneys, the consequence of a kidney tumor is clearly different for papers using KiTs19. As seen in Figure 8, the V-Net and U-Net are the optimal configurations. The comparison using KiTs21 and the other dataset are shown in Figure 9. Table 7 shows a summary of results using other metrics.

### 5.2. Deep Learning

The DL approach is trained to do segmentation on a pixel-by-pixel or region-by-region basis. To begin, features are retrieved to distinguish the lesion from the background, and different lesion characteristics can be integrated via feature extraction. However, due to the lengthy training period and potential for over-training, test images should originate from the same platform as the training images. DL techniques require a huge quantity of training data to perform effectively on previously unknown materials. However, this presents several difficulties in the medical area. First, annotating even a single CT volume requires a substantial amount of time for a well-trained radiologist. Additionally, the work is prone to intra- and inter-rater variability. As a result, all annotations are authorized by an excessive number of expert neuroradiologists [139].

A significant amount of effort is spent obtaining permission, and fortunately, deep convolutional neural networks have demonstrated exceptional performance in a variety of computer vision applications. These networks, however, rely largely on massive amounts of data to avoid overfitting. Overfitting is a term that refers to the process by which a network learns a function with a very large variance in order to predict the training data properly. Regrettably, many application fields, such as medical image analysis, lack access to huge data [140]. Because the unavailability of large-scale datasets limits the potential of deep learning models, researchers have turned to data augmentation as a quick fix for the data issues described above. Other recent studies have examined unsupervised learning [141]. Numerous augmentations have been proposed, most of which can be categorized as data warping or oversampling techniques. Data augmentation has a very bright future. The potential for using search algorithms that combine data warping and oversampling techniques is immense. Deep neural networks’ layered architecture provides numerous chances for data enhancement [81].

Regarding hardware requirements while training a model with huge datasets, DL techniques demand massive computations processes. Due to the fact that the advantage of a GPU over a CPU rises with the scale of the computations, the GPU is mostly used to successfully optimize processes. Thus, GPU hardware is required to perform successfully with deep learning training. As a result, DL is more reliant on high-performance machines equipped with GPUs than on traditional machine learning approaches [142]. Additionally, the use of 3D deep learning models significantly increases the computational and memory requirements. DL uses software libraries to define and train deep neural networks in a parallel or distributed manner while leveraging multi-core or multi-GPU environments. At the moment, researchers are constrained by the amount of GPU memory available to them (typically 12 gigabytes). As a result, batch sizes and model complexity are constrained by the amount of memory available [21].

The performance of kidney tumor segmentation algorithms have continued to improve in recent years as more training data has been available and more sophisticated CNN architectures and training techniques have been used. Their robustness, however, continues to fall behind expert performance [21]. Interestingly, single U-Net [83] based models [143] continue to perform well, corroborating the assertion that “a well-trained U-net is difficult to surpass” [144]. As a result, concerns such as properly analyzing 3D slice data and compressing the model as the number of network parameters rises, must be addressed in the future. Additionally, because of the scarcity of medical imaging data, researchers must consider how to avoid over tagging. Although the features of individual tumors vary, there are several similarities between them. However, no successful study presently aims to unify segmentation algorithms and transfer learning across tumor types [88]. The examined literature demonstrates that careful initialization of hyper-parameters, pre-processing techniques, sophisticated training schemes, and addressing the class imbalance problem contribute significantly to the accuracy and resilience of segmentation algorithms.

## 6. Conclusions and Future Work

This paper discusses ways of segmenting kidneys and kidney tumors using deep learning and building blocks, as well as state-of-the-art approaches and implementation tools. The existing techniques serve two purposes: segmenting tumors correctly and compensating for the lack of training data. Based on adequate training data, DL is capable of adequately segmenting kidney tumors. With proper pre-processing, weight initialization, sophisticated training schemes, segmentation with unambiguous borders, and obtaining additional information for pixel classification, ensemble approaches and U-Net-based models have significant potential for improving the state-of-the-art. The absence of a large-scale medical training dataset is a primary reason for the poor performance of many segmentation algorithms. As a starting point for future development, overall, kidney and renal tumor segmentation challenges have been met with great success. It received a large number of submissions and continues to be a significant and hard benchmark for 3D segmentation. However, extending the use of these systems outside the sampled population for the test set would be desirable since it was obtained from individuals who shared the same geographic region and healthcare system and a multi-institutional cohort with a prospectively generated test set. Additional imaging modalities, such as magnetic resonance imaging (MRI) or contrast-enhanced ultrasound (CEUS), may be employed to increase the diagnostic algorithm’s accuracy when CT alone is used. Future research on DL designs, particularly in the realm of medical imaging, should avoid complicated architectures. Future research should focus on reducing the training time for deep learning models. To produce more successful models that can be utilized in various disciplines, it is required to simplify the systems to which the models may be applied and minimize their complexity.

## Figures and Tables

**Table 1:** Evaluation metric for segmentation.

Metric	Equation	Description
True Positive rate (TP)	TPR = Sensitivity = Recall = $\frac{TP}{\left(TP+NF\right)}$	True positive rate, the proportion of true positives or successes that is accurately detected, is calculated as true positive rate, also known as sensitivity [98].
True Negative rate (TN)	TPR = Sensitivity = Recall = $\frac{TP}{\left(TP+NF\right)}$	The true negative rate, also known as specificity, is the true negative rate. Is there a the chance that a non-diseased will the person be classified as negative by the test? It demonstrates the test’s sensitivity in identifying the absence of illness [99].
False-positive rate (FP)	FP = $\frac{FP}{\left(FP+TN\right)}$	The false-positive rate refers to the percentage of mistakenly classified as positive or successful but as negative [98].
Dice Similarity Coefficient (DSC)	DS = 2× $\frac{|S_{m}⋂S_{a}|}{|S_{m}+S_{a}|}$	The binary mask produced by the domain experts’ manual segmentation corresponds to the binary mask produced by the suggested approach. DC must be close to unity to guarantee that the manually drawn region corresponds to the segmented result correctly [99].
Jaccard Index JI	JI = $\frac{|S_{m}⋂S_{a}|}{|S_{m}+S_{a}|}$	The Jaccard Index (JI) was used to compare the statistical similarity of regions segmented using a computational approach to hand delineations [100,101].
Accuracy	Accuracy = $\frac{\left(TP+TN\right)}{\left(TP+FP+NF+TN\right)}$	The correct predictions produced by the prediction model across all suitable forecasts completed are referred to as the model’s accuracy [102].
Precision	Precision = $\frac{TP}{\left(TP+FP\right)}$	The number of correct positive scores divided by the number of positive scores anticipated by the classification algorithm is the positive predictive value, or precision [103].
Sørensen–Dice	DSC = $\frac{2TP}{2TP+FP+FN}$	This coefficient: Indicates the extent to which segmented and reference volumes overlap in mm3. (1 for ideal segmentation, 0 for the worst-case scenario) When applied to Boolean data, the terms true positive (TP), false positive (FP), and false negative (FN) are used (FN), As in this case [47].

**Table 2:** Summary of commonly used public datasets for kidney and kidney tumor segmentation.

References	Total	Training Data	Validation	Testing Data
KITS19 [39,40,44,59,72]	300	210	-	90
KITS19 [12]	210	134	34	42
KITS19 [61]	300	240	-	60
KITS19 [62]	300	190	20	90
KITS19 [70]	300	240	30	30
KITS19 [41,58]	300	168	42	90
KITS21 [55,63,65,69]	300	240	-	60
OTHER [50]	113	70	23	20
OTHER [54]	140	90	-	50

**Table 3:** Summary of the benefits and drawbacks of various segmentation techniques.

Type of Segmentation	Reproducibility	Time	Interactivity	Complexity of Implementation
Manual Segmentation	Good	Too long	Bad	Easy
Semi-Automatic Segmentation	Good	Long	Not bad	Easy
Fully Automatic Segmentation	Good	Short	Good	Hard
Semantic Segmentation	Good	Short	Good	Hard

**Table 4:** Overview of Deep Learning methods for kidney tumor segmentation: PA = Pixel accuracy, SS = Specificity-Sensitivity, KD = Kidneys Dice, TD = Tumor Dice, CD = Composite Dice, DSC = Dice = Dice similarity coefficient DSC, CD = Centroid distance, HD = Hausdorff distance, AC = Accuracy, SGD = Stochastic gradient descent, SD = Surface Dice, BN = Batch Normalization, IN = Instance Normalization, SGD = Stochastic Gradient Descent, IOU = Intersection over Union.

Reference	Input	Regulization	Activation	Loss	Optimizer
U-Net Architecture					
[127]	3D			Dice	Decathalon
[128]	3D	BN, Depthwise, Weight Pruning	RELU	Mean IoU, AC	Adam
[49]	3D,2D		RELU	Dice	SGD
[129]	3D			Dice	Adam
[60]	3D		Leaky ReLU	Dice	Adam
[53]	3D			Dice, SD	Adam
[12]	3D	IN		Dice	Adam
[130]	3D,2D	BN	ReLU	Dice	Adam
[131]		BN	RELU	IOU	
[56]	3D		ReLU	Dice	Adam
[55]	3D	BN	ReLU	Dice	Adam
[69]	3D		ReLU	Dice	Adam
[68]	3D	Batch norm	ReLU	Dice	Adam
[67]	3D	BN	ReLU	Dice	Adam
Cascaded Architecture					
[63]	2.5D	BN	ReLU, conv	Dice	Adam
[41]	3D	BN	SE-Net	Dice	Adam
[51]		Dropout	RELU	Dice, CD, HD	
[59]	3D	BN	ReLUs	Dice	Adam
[65]	2D	BN	RELU, LeakyRelu	Dice	
3D U-Net Architecture					
[39]	3D		RELU	Dice	SDG
[66]	3D		RELU	Dice	
[132]	3D	BN	ReLU	Dice	
[57]	3D	BN	ReLU	Dice	Adam
Boundary-Aware Architecture					
[44]	3D	BN	RELU	Dice	Adam
[5]	3D		RELU	KD, TD, CD	Adam
V- Net Architecture					
[40]				Dice	Adam
[19]	3D			Dice	
Ensemble Architecture					
[47]	2D		RELU	Dice	
[64]				Dice	Adam

**Table 5:** Overview of Deep Learning methods for kidney tumor segmentation on other architecture.

Reference	Architecture	Input	Regulization	Activation	Loss	Optimizer
[50]	MB- FSGAN	3D	BN	RELU	PA, Dice, SS	RMSProp, Adam
[58]	U-Net, AlexNet	2D	BN	RELU	Dsc, Jaccard index, AC, SS	Adam
[133]	Modified CNN	2D	Weight Decay		Dice	
[61]	EG- CNN	3D		RELU	Dice	Adam
[54]	FCN	3D	L2		Dice	SDG
[46]	RAU- Net	3D			Dice	SDG
[62]	multi- stage U-Net	2.5D	BN	pre- activation	Dice	Adam
[52]	CTumor GAN	3D	BN, Dropout	RELU	Dice, Jaccard index, SS	Adam
[73]	nnU-Net	3D	IN		Dice, Jaccard, Ac, Precision, Recall, Hausdorff	Adam
[48]	FPN (CNN)	2D			Dice	
[45]	CNN	2D,3D			Dice	
[71]	3D SEAU -Net	3D	BN		Dice	
[134]	DeepLab v3+	3D	BN	RELU	Dice	Adam

**Table 6:** A summary of results on KiTs 2019, KiTs2021, and another dataset.

Reference	Kidneys Dice	Tumor Dice	Composite Dice
KiTS19			
[61]	0.965	0.835	0.900
[60]	0.967	0.845	0.906
[39]	0.974	0.851	0.912
[129]	0.97	0.32	
[59]	0.974	0.831	0.902
[12]	0.969	0.805	0.887
[62]	0.98	0.73	0.855
[40]	0.977	0.865	0.921
[19]	0.973	0.817	
[70]	0.978	0.868	0.923
[5]	0.974	0.810	0.892
[134]	0.872	0.384	
[41]	0.968	0.743	0.856
[47]	0.949	0.601	
[44]	0.970	0.834	0.902
[46]	0.960	0.770	
[66]	0.930	0.570	
[64]	0.968	0.750	
[45]	0.964	0.674	
[71]	0.924	0.743	
[72]	0.852		
KiTS21			
[63]	0.943	0.778	
[49]	0.975	0.881	0.871
[53]	0.923	0.553	
[65]	0.934	0.643	
[67]	0.96	0.81	
[68]			0.654
[69]	0.916	0.541	
[55]	0.937	0.750	825
[56]	0.90	0.39	
Other Dataset			
[50]			0.859
[54]	0.923	0.826	0.875
[51]			0.925

**Table 7:** A summary of results using other metrics.

Reference	Sensitivity	Specificity	Jaccard	Accuracy	Hausdorff
[12]			0.716	0.99	33.469
[70]	0.913	0.914			5.10
[50]	0.862	0.894		0.957	
[72]	0.842	0.998	0.756	0.997	18.39

## Data Availability

Data available in publicly accessible repositories.
